# Maize pollen diet enhances malaria mosquito longevity and infectivity to *Plasmodium* parasites in Ethiopia

**DOI:** 10.1038/s41598-023-41826-7

**Published:** 2023-09-02

**Authors:** Shilimat Ayele, Teklu Wegayehu, Nigatu Eligo, Girum Tamiru, Bernt Lindtjørn, Fekadu Massebo

**Affiliations:** 1 Department of Biology, Wachemo University, Hossana, Ethiopia; 2 Department of Biology, Arba Minch University, Arba Minch, Ethiopia; 3 Centre for International Health, University of Bergen, Bergen, Norway

**Keywords:** Entomology, Malaria

## Abstract

Although larval diet quality may affect adult mosquito fitness, its impact on parasite development is scarce. Plant pollen from *Zea mays*, *Typha latifolia*, and *Prosopis juliflora* was ultraviolet-sterilized and examined for effects on larval development, pupation rate, adult mosquito longevity, survival and infectivity. The control larvae were fed Tetramin fish food as a comparator food. Four treatment and two control groups were used for each pollen diet, and each experimental tray had 25 larvae. Female *An. arabiensis* were starved overnight and exposed to infectious blood using a membrane-feeding system. The Kaplan–Meier curves and log-rank test were used for analysis. The *Z. mays* pollen diet increased malaria mosquito survival and pupation rate (91.3%) and adult emergence (85%). *Zea mays* and Tetramin fish food had comparable adulthood development times. Adults who emerged from larvae fed *Z. mays* pollen had the longest average wing length (3.72 mm) and were more permissive to *P. vivax* (45%) and *P. falciparum* (27.5%). They also survived longer after feeding on infectious blood and had the highest number of *P. vivax* oocysts. *Zea mays* pollen improved larval development, adult mosquito longevity, survival and infectivity to *Plasmodium*. Our findings suggest that malaria transmission in *Z. mays* growing villages should be monitored.

## Introduction

*Plasmodium falciparum* and *P. vivax* are the two dominant human malaria parasites in Ethiopia, and the principal vector is *Anopheles arabiensis.* Some mosquito species are efficient and highly permissive to the parasite to complete the cycle and predominantly bite humans to increasing the parasite transmissibility^1^. There is also a link between the intensity of malaria transmission and the biology of the mosquito vectors. For instance, the quality of larval food may have an impact on adult life histories, including adult survival, the number of emerging adults, and the development of parasites^2^. Female *Anopheles* mosquitoes usually select breeding habitats suitable for the survival and development of immature stages. The pollen (fine to a coarse powdery substance comprising pollen grain) from cultivated and wild grasses supplement the natural food for *Anopheles* larvae in the breeding habitats and might support the development and increase malaria transmission^3,4^. For example, maize cultivation is mostly claimed to increase malaria transmission^5^. The odour from the maize pollen also has lured gravid malaria mosquitoes to the oviposition site^6^. Moreover, nutrition during the larval stage determines the size and metabolic reserves of adult mosquitoes^4,7^.

There are, however, controversial results with regard to the plant-vector interaction. For example, a study by Muller and his colleagues^8^ reported higher risk of malaria infection during the flowering season of an invasive plant species *Prosopis juliflora*. When the flowering branches of this plant were removed, the age structure of the malaria mosquitoes changed, and the number of older malaria mosquitoes was substantially reduced. It might be linked to the sugar that flowering plants provide adult mosquitoes as a source of energy, which can influence their age. The pollen of some plants is more nutritious than others. However, several plants may inhibit disease transmission by affecting vectors in their breeding habitats because of the presence of toxic constituents. For example, *Cyperus papyrus* and *Phragmites australis* contains chemicals that can kill the larval stages in breeding habitats^9^. Some plants have very attractive flowers for mosquitoes and some others have toxic sugar baits^10^.

Existing knowledge on the interaction of malaria vectors and plants with respect to parasite transmission is scarce. *Zea mays* (a maize specimen) is the most common crop in the lowlands of Gamo zone where malaria is also endemic^11^. The crop is cultivated all year round. The shores of the Lake Abaya and Chamo are predominantly covered by *T. latifolia.* The shores of the two lakes are also ideal breeding sites for the primary and secondary malaria vectors^11,12^*.* The shrub *P. juliflora* is also a very common plant species in the region. While there is little evidence about the interaction between malaria vectors and plants, effective control requires a contextual understanding of the bio-ecology of malaria vector-plant interaction. However, in Ethiopia, there is limited understanding of the impact of pollen on early stages and its long-term effect on adult fitness and susceptibility to the parasite. In light of these factors, the purpose of this study was to demonstrate whether pollen from *T. latifolia*, *Z. mays* and *P. juliflora* enhances *An. arabiensis* larval development and survivorship, pupation rate, adult size, longevity of malaria vector, survival and infection to *Plasmodium* parasites.

## Materials and methods

### Study area description

*Anopheles* larval sampling was made from the potential breeding habitats in the Arba Minch area, located in the southern Ethiopian Rift Valley system. The climate is hot and humid which is favourable for mosquito breeding and malaria transmission. The average maximum temperature is 30 °C, while the average minimum is 17 °C. The rainfall distribution is bimodal and mostly occurring in March to May and September to November. The annual rainfall average is around 900 mm. The major cash crops cultivated in the area include bananas, maize and mango using both rain and irrigation schemes. In the rainy seasons, the water pools in ditches along the roads, farmlands, tire track pooled water, and marshy areas are the potential breeding sites for *Anopheles* mosquitoes.

Malaria is a common health problem in the area^11,13^ and is more prevalent after the end of the rainy season due to the potential breeding sites^11^. *Plasmodium falciparum* and *P. vivax* are the two common species^14,15^.

### Study design

**Figure 1:**
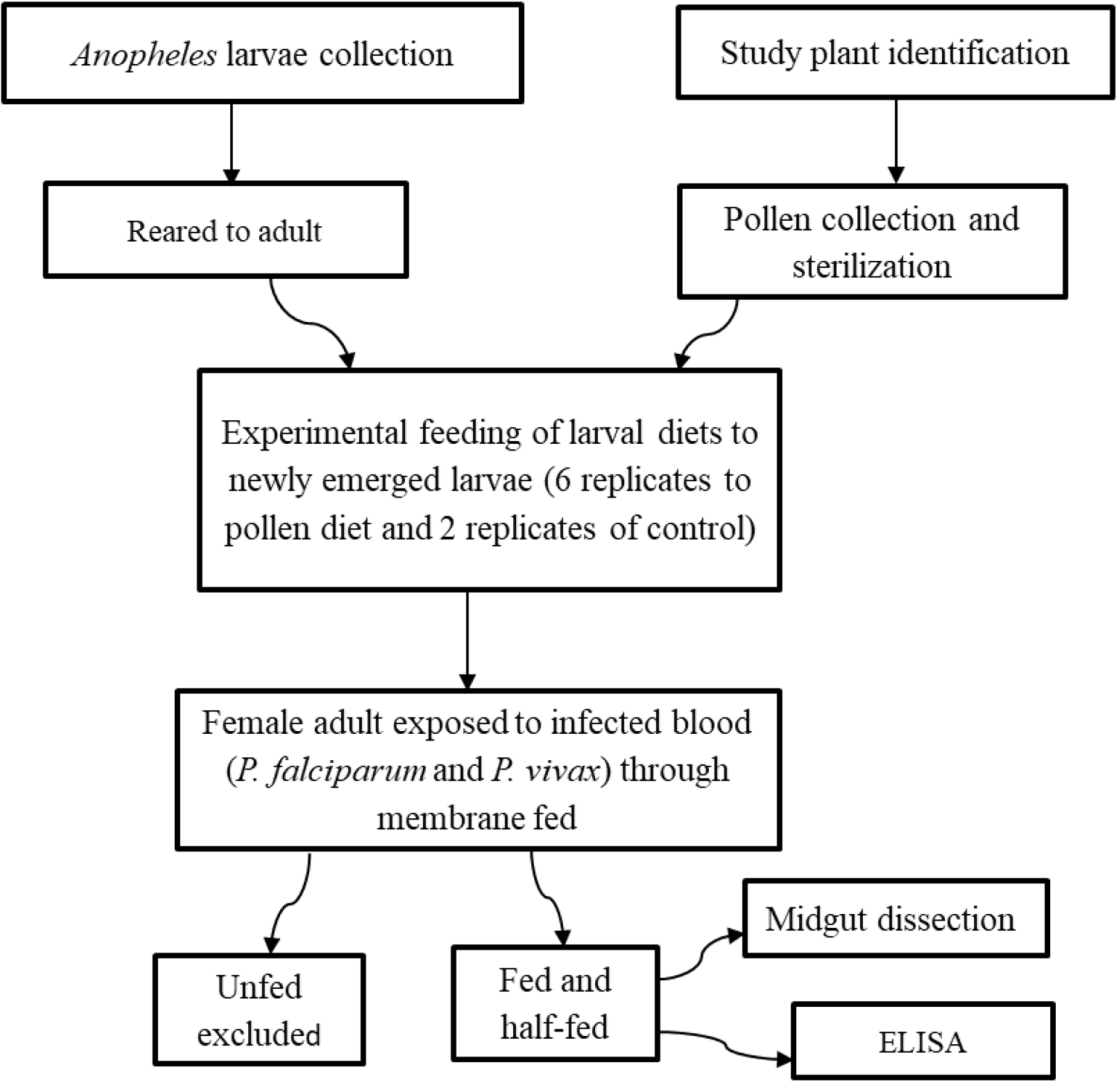
The study flow chart.

This experiment aimed to assess the effect of various plant pollen diets on key vectorial aspects of *An. arabiensis*. Newly emerged larvae were provided with equal quantity of pollen from *T. latifolia*, *Z. mays*, *P. juliflora* and standard larval food Tetramin fish food (Cichlid Sticks; Tetra, Maidenhead Aquatics, Leicester, UK). The emerged adults were supplied with10% sugar solution until ready for the infection experiments^16^. Then, 2–3 days old female *Anopheles* were starved overnight and provided with *P. vivax* and *P. falciparum* parasite infected blood though membrane feeding apparatus (Fig. 1).

### Study plants

**Figure 2:**
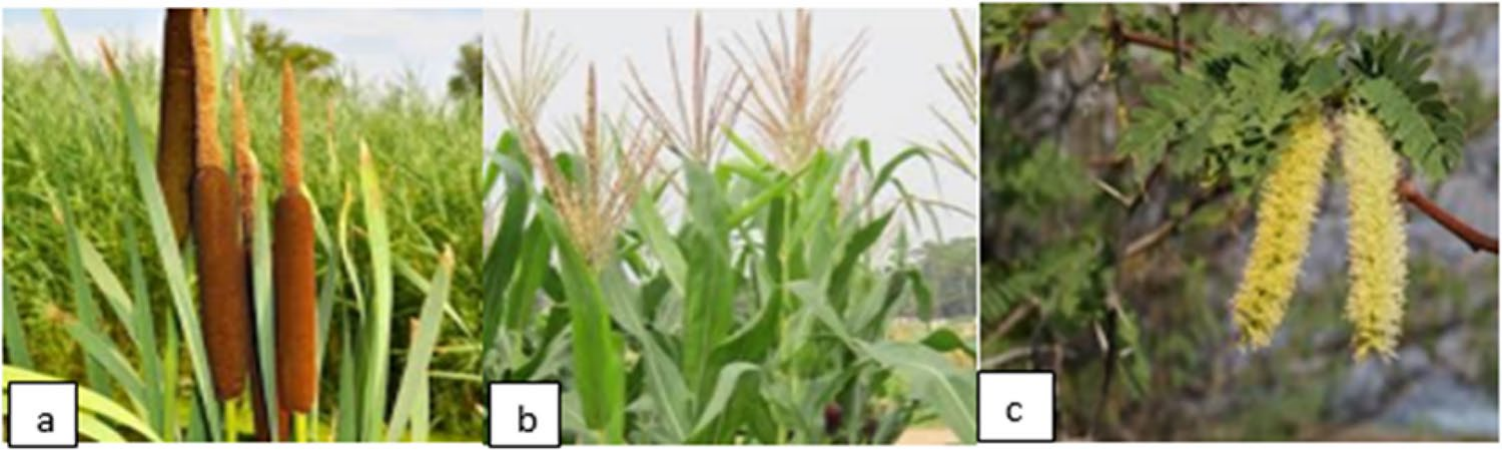
Study plants (**a**) *T. latifolia*, (**b**) *Z. mays*, (**c**) *P. juliflora.*

These plants (Fig. 2) were selected based on the scientific evidences documented in relation to the malaria mosquitoes either against or in favour of larval and adult stages^5,7^. *Typha latifolia* (common cattail) is a perennial herbaceous plant in the genus *Typha* and among the most common of all aquatic plants. It is an "obligate wetland" species found close to water bodies. This plant is among the wind-pollinated plants that produces large quantities of pollen. *Prosopis juliflora* is a shrub or small tree in the family Fabaceae. It is an evergreen and fast-growing invasive species in arid and semi-arid areas. The flowers of *P. juliflora* are self-incompatible, entomophilous (insect pollinating), depending on insects for seed dispersal. It produces many seeds and is tolerant of a wide range of climatic regimes and soil types which have contributed to making *P. juliflora* one of the worst invasive alien plants. The leafy stalk of *Z. mays* (maize) produces pollen inflorescences and separate ovuliferous inflorescences called ears that yield seeds, which are fruits and its pollen, are produced entirely in the staminate inflorescence that is wind pollinated. The male flower matures earlier than the female flower. The plant specimens were collected from their typical habitat by field press and brought to the Arba Minch University Botany laboratory for further confirmation by expertise.

### Pollen collection

Pollen of *T. latifolia* was collected in the shore of the Lake Abaya, *P. juliflora* from the shrub dominated area of Lante (local place in Arba Minch area) and the pollen of *Z. mays* L. (BH660 cultivar) from the farmland of Lemat during the maize flowering season. The pollens were collected by covering the mature flower with a paper bag and shaking. The collected pollen was transferred to vials and sterilized within 24 h of collection using UV light (10850 Harry Hines Blvd Suite 155, Dallas, TX 75220) overnight for 12 h and store on silica gel until used for feeding experiments.

### Pollen nutrient analysis and measuring grain size

The C and N concentration of pollen diets were determined by Walkley–Black and Kjeldahl methods in Arba Minch University analytical chemistry laboratory. Approximately 5 mg of sample of pollen from each plant was taken and processed based on the standard protocol developed by Roulston et al^17^. Thereafter, the C: N ratio of the pollen was determined by %C/%N × 6.25^17^. The pollen grain sizes were measured according to Roulston et al^17^. Pollen grains were mounted in paraffin oil and measured at 400 × with an ocular micrometer. Polar axis measurements were taken for all pollen grains. Volumes were calculated using volume equations for spheres (4/3πp3). For the purpose of calculating grain volume, ten randomly selected grains per slide were measured, and these values were averaged.

### Mosquito larvae collection and rearing

*Anopheles* larvae were collected from the potential breeding habitats in the Arba Minch area and reared in Arba Minch University Medical Entomology and Vector Control laboratory. Swamps along the Lake Abaya shoreline and water holding areas at construction sites were the most common breeding habitats where the larvae were collected. Additionally, there were a few small bodies of water gathered alongside ditches and rivers that were *Anopheles* larvae-positive. The rearing room was maintained at temperature between 25 and 27 °C and relative humidity between 72 and 80%. The larvae rearing trays were inspected twice a day for the presence of pupae, and if present they were placed in adult cages. The larvae collected from natural habitats were not employed in this experiment due to the range of habitats and food sources.

The newly emerging adults were provided with 10% sterilized sugar solution. Then, they were allowed feed on live rabbit at 3- to 4-days interval and serving as ‘mother’ cages. The egg dish was placed in the cage one day after the adult fed on blood, and eggs were transferred to the larvae tray for hatching. Using these larvae, an experiment was conducted.

The female adult mosquitoes in the mother cage were morphologically identified to the *An. gambiae* complex, and a sub-sample (150) was tested for *An. gambiae* complex by polymerase chain reaction (PCR) in the Medical Entomology laboratory at Arba Minch University following the protocol of Scott et al^18^. To mention in brief, legs or wings were used for DNA extraction using the NucleoSpin Tissue kit (Macherey Nagel, Germany) according to the manufacturers’ instructions. The species-specific nucleotide sequences of the rDNA intergenic spacers (IGS) were targeted for *An. gambiae* complex as described by the protocol mention above. The PCR products were visualized on a 1% agarose gel using a 100 bp ladder (Promega, The Netherlands) and species identification was done based on the band height. The entire tested *An. gambiae* complex was identified as *An. arabiensis* (hereafter the target mosquito species will be *An. arabiensis*).

### Larval development and survival analyses

After 12 h of hatching, 200 unfed first instar larvae were counted manually and randomly apportioned to larval trays of 10 × 25 cm and 8 cm high filled with one litter of distilled water. Effective dose of pollen diets and TetraMin fish food was determined by optimizing 0.15, 0.3 and 0.6 mg on relative adulthood survival. Then, 0.3 mg was established as the effective dose of each pollen diet and Tetramin fish food.

Six replicates each with 25 larvae for each of the plant pollen diet and two as control group were used. The experiment was repeated three times for each pollen diet and control group of TetraMin fish, using different mosquito batches from the field. A total of 450 larvae were treated for each diet and 150 for each experimental term. For the first and the second instar larvae, the diet was offered only once a day, for the third and fourth, it was twice a day based on procedure of Kivuyo et al^19^. In each replicate, the larval development and survival was recorded twice a day (01:00 and18:00) and dead individuals were counted and removed. Continuous inspection of the number of larvae surviving, pupating, and emerging adults were recorded.

$$ {\text{Survival}}\;{\text{rate}}\;{\text{of}}\;{\text{each}}\;{\text{day}}\;\left( {{\text{s}}_{{\text{j}}} } \right) = \frac{{{1} - {\text{dead}}\;{\text{larvae}}\;{\text{at}}\;{\text{day}}\;{\text{j}}}}{{{\text{number}}\;{\text{of}}\;{\text{survivors}}\;{\text{at}}\;{\text{day}}\;{\text{j}}}} $$

$$ {\text{Survival}}\;{\text{rate}}\;{\text{total}}\;\left( {\text{S}} \right) = {\text{s}}_{{1}} + {\text{s}}_{{2}} \cdots + {\text{sj}} $$

where s_1_ the no. of larvae at the begning of day 1, s_2_ the no. of larvae at the begning of day 2.

### Measuring mosquito size/wing length

The same batches and same ages of 30 female adult *An. arabiensis* were collected from each pollen diet and TetraMin fish food. They were killed by freezing and, then one wing of each mosquito glued onto a slide. Its length from the distal end of the alula to the tip, excluding the fringe scales, was measured with stage graticule micrometer (ruler etched in side) to examine the effect of larval diet on wing length of adult *An. arabiensis*.

### Patient screening for gametocyte stage

Microscopically positive patients with *P. vivax* and *P. falciparum* gametocytes were enrolled. The gametocyte detection was done by standard finger prick and Giemsa stained blood smears from thick blood smear under oil immersion microscopy by experienced laboratory technicians. The gametocyte positive patients were asked to provide approximately 3 ml of blood for membrane feeding prior to antimalarial treatment. Blood gametocyte density was estimated microscopically by converting the total number of gametocytes in 1000 leukocytes to 8000 leukocytes/μl. Screening of gametocyte carriers was synchronized into mosquito rearing processes and thus always performed on the same day with the experimental infection. Patients with mixed infection were excluded.

### Membrane blood feeding

The same batch of 2 to 3-day-old female *An. arabiensis* were starved overnight and kept for experimental infection through membrane feeding. A total of 1280 female *An. arabiensis* were allowed to feed on *P. vivax* positive blood in four membrane feeding experiments, and another 1280 were exposed to *P. falciparum* positive blood in four membrane feeding experiments. In each feeding experiment, 320 *An. arabiensis* were exposed in 8 paper cups each with 40 mosquitoes.

To keep the blood warm and the parasites alive, a circulation water bath was used, and its temperature was set to 37 °C. After carefully pipetting a *Plasmodium*-positive blood meal into the feeder's neck, it was then allowed to feed simultaneously for 30 min in the dark. Unfed mosquitoes were discarded after carefully inspecting them for abdominal conditions. The fed *An. arabiensis* were kept in the paper cups and provide with 10% sugar solution under normal insectary condition (27 °C ± 2 and 70 ± 10% relative humidity). *Anopheles arabiensis* were kept under sugar for eight days for *P. vivax* and twelve days for *P. falciparum* oocyst detection. The cups were put inside the small cage as an extra layer of protection in case the mosquitoes that feed on the infectious blood left. The membrane was removed carefully and soaked in 10% bleach to decontaminate and rinse with water.

### Dissection to oocyst stage and sporozoite detection

Randomly 40 mosquitoes were taken from each pollen type replicate cages on day 7 post-infection for *P. vivax* and on day 12 post infection for *P. falciparum.* A total of 120 female mosquitoes were dissected for *P. vivax* and *P. falciparum*. For each of the dissection sessions, the infection rate (infected mosquitoes/total mosquitoes dissected in each feeding (%) was recorded, and intensity of infection (oocyst load/total infected mosquitoes dissected in each feeding) were determined. Non-infected mosquitoes were omitted from the calculation.

The infective stage of the *Plasmodium* parasite was detected by circum-sporozoite protein (CSP)-ELISA using the kits specific for *P. vivax*-210, *P. vivax*-247 and *P. falciparum* following standard instruction^19^. A total of 104 and 35 *An. arabiensis* were tested for CSPs *P. vivax* and *P. falciparum,* respectively.

### Data analysis

In order to conduct a survival analysis, data from the experiment on the impact of diets on larval survival were transformed to count-time data. Kaplan–Meier curves were used to compare diet-specific survival pattern for each group. Comparison of survival rate in each larval diet to that of TetraMin Fish food was performed using log rank test. The Analysis of Variance (ANOVA) test was conducted to evaluate how larval diets impacted the mean pupation rate, adult emergence rate, and wing lengths. Statistical testing was performed using the mean of each pollen diet, taking into account the origin of the mosquitoes in each batch. Wald’s test was used to determine significant differences between diets on the vectorial parameters of *An. arabiensis* based on χ^2^ and *P*-values.

The proportion of female adult mosquitoes that survived after infected blood meal was determined by counting the number of mosquitoes on their oocyst and sporozoite stage across all larval diets. The median number of oocyst per mosquito mid-gut was compared using a Kruskal–Wallis test, followed by a Dunn's post-hoc test. The prevalence of sporozoite infection, or percentage of mosquitoes reaching to infective stage, was compared with a Fisher's exact test.

All statistical analysis performed using GraphPad Prism 5 (Version 5.01). Significance was considered at 5% level of confidence. Single factor experimental design was employed to assess the impact of pollen of some selected plants on the larval survival, pupation, adult emergence, and vector infectivity to malaria parasites. Survival rate of each treatment case was calculated as survival rate of each day.

### Ethical consideration

This study was reviewed and approved by the Institutional Review Board of Arba Minch University (No: CMHS/12036564/111). After briefing the objective of the study, consent was obtained from the study participants. Participants in the study who tested positive for *Plasmodium* received free treatment in accordance with the national malaria treatment guidelines. The CSP-ELISA test was performed in accordance with standard protocol^19^. All methods were performed in accordance with the standard protocols and procedures^19^. Other plant pollens, aside from maize pollen, were collected with the local administration approval in open spaces like the lakeshore and by the side of the road. Through permission of farm owners, the maize pollen was collected on farmland. Despite the fact that only the pollen grains were gathered for the experiment, plant specimens were also gathered in order to be identified by senior botanists before collection. The voucher specimens of the plants (*P. juliflora* and *T. latifolia*) have been deposited in a publicly available botanical herbarium at Arba Minch University.

## Results

### Pollen nutrient content and size

The pollen grain volume was significantly different between plants with higher pollen volume of *Z. mays* (mean diameter 71.2 ± 1.66 µm) compared to *P*. *juliflora* (48.1 ± 0.56 µm) and *T*. *latifolia* (25.5 ± 0.32 µm) (F = 1033, DF = 3, *P* < 0.0001).

The C: N ratio of pollen differed between test plants with the pollen C: N ratio of *T. latifolia* > *Z. mays* > *P. juliflora*. *Typha latifolia* had a higher proportion of carbon (52%), compared with the two other species *Z. mays* (44%), and *P. juliflora* (32%). The higher proportion of nitrogen was belonging to *Z. mays* (31%) followed by *T. latifolia* (28%) and *P. juliflora* (18%).

### Larval diet impacts on larval survival

**Figure 3:**
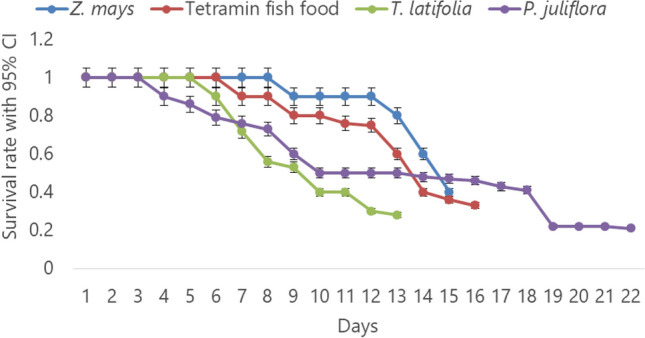
*Anopheles arabiensis* larvae survivorship and duration of larval development in larval diet of pollens of *T. latifolia*, *Z. mays* and *P. juliflora* and TetraMin fish food.

Survivorship of the larval instars was found to be diet-dependent. The overall larval survival differed significantly among the pollen diets tested (F = 23, DF = 3, *P* < 0 0.0001). The survival rate of larvae fed on the *Z. mays* pollen was 100% until day 8 and slowly declined with the minimum survival rate of 0.4 at day 15. In the follow-up period, the likelihood of survival for larvae fed on diets of *Z. mays* pollen was 0.89, followed by T. latifolia (0.71). The survivorship of larvae due to *P. juliflora* pollen diet was found to be the poorest (highest larval death) with 0.51 probability of survival during the follow-up period. The group of *An. arabiensis* larvae supplied with *T. latifolia* pollen had accelerated development of larval instars, while those supplied with the *P. juliflora* pollen diet experienced extended larval period (Fig. 3).

### Effect of larval diets on pupation rate and duration

The effect of pollen diets on the larval average time to complete the larval stage and change to the pupae showed significant differences between the diets (χ^2^ = 12.8, DF = 3, *P* < 0.0001). *Anopheles arabiensis* pupation was faster on *T. latifolia*, it took 2-to-3-days since the starting day of pupation followed by Tetramin fish and *Z. mays*. Larval instars took longer time to develop into pupae when larvae supplied with *P. juliflora* pollen diet 5-to-11 days (Table 1).

**Table1:** The impact of different larval diets on *An. arabiensis* larvae pupation rate and duration of pupation.

Larval diet	Average no. of pupae/150 larvae (%)	Average no. of pupae/day	Starting day of pupation	Average time to complete pupation since starting day of pupation
*T. latifolia*	111 (74)	5.4	5	2-to-3-days
*Z. mays*	137 (91.3)	12	8	4-to-5-days
*P. juliflora*	96 (60.9)	1.95	10	5-to-11-days
Tetramin fish food	128 (85.3)	7.5	6	3-to-4-days

### Adult emergence rate

**Figure 4:**
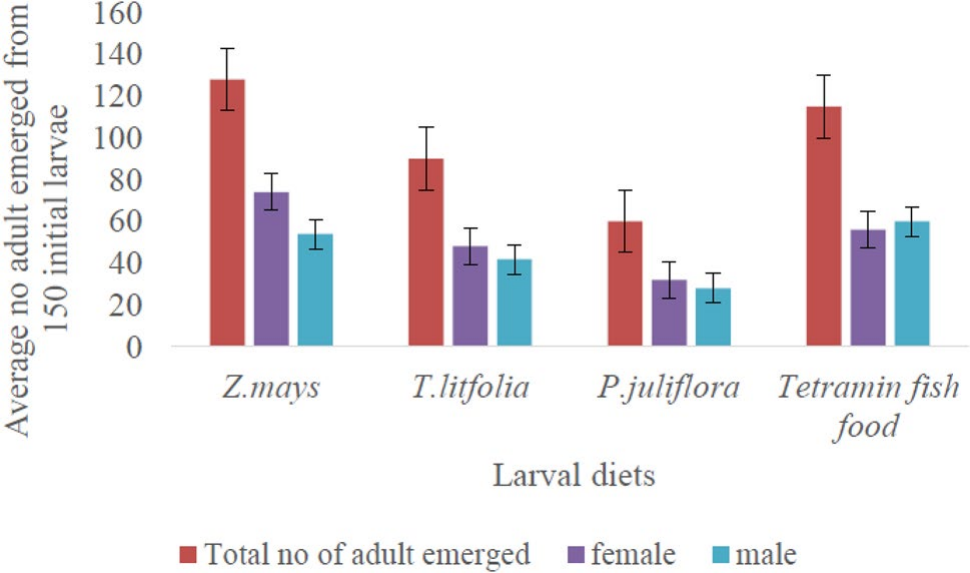
The number adults emerged from initial number (n = 150) of larvae in different larval diets.

A significant difference was observed between larval diets from different plants on adult emergence rate (x^2^ = 17.3, DF = 3, *P* < 0.0001). The highest adult emergence rate was documented in *Z. mays* diet (*P* < 0.05) followed by TetraMin fish food and *T. latifolia*. The number of adults that emerged in *P. juliflora* was lower compared to the three other diets (Fig. 4). Moreover, high mortality of adult mosquitoes was also documented on this diet. But there was no significant deviation in time needed to reach adulthood in either of the diet (*P* > 0.05), mostly, emerged with in 48 h after pupation. More females emerged from larvae fed on *Z. mays* pollen, compared to other diet sources but not significantly different (*P* > 0.05).

### Larval diet effects on wing length

**Figure 5:**
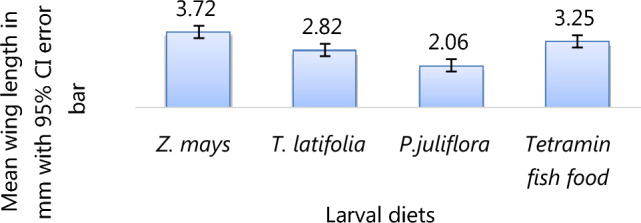
The effect of larval diets on wing length (WL) of adult female *A. Arabiensis.*

Wing length was significantly affected by the type of larval diet (χ^2^ = 69, DF = 39, p < 0.0001). The female *An. arabiensis* emerged from larvae fed on *Z. mays* possessing bigger wings (mean WL = 3.72 mm) than females that fed on Tetramin fish (mean WL = 3.25 mm) and *T. latifolia* (mean WL = 2.82 mm). The overall mean wing length was 2.9 mm, with a range of 2.06 mm to 3.72 mm. The *An. arabiensis* that were reared on *P. juliflora* pollen had the smallest wing size (mean WL = 2.06 mm) (Fig. 5).

### Larval diet impacts on adult survival and oocyst density

**Figure 6:**
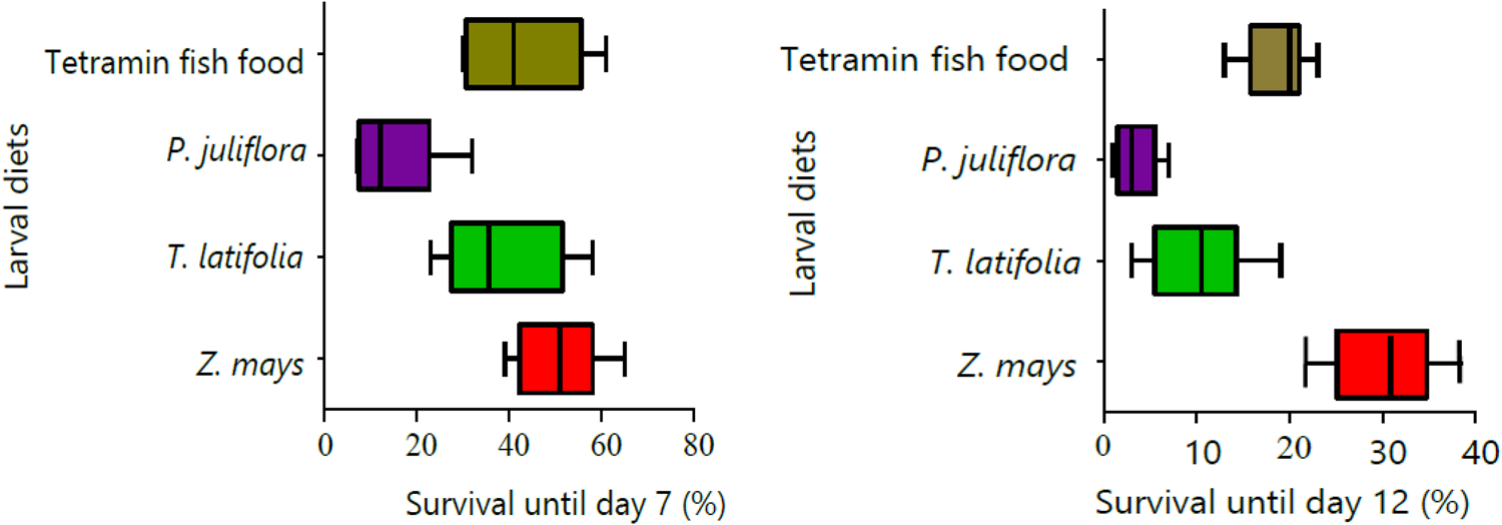
The percentage of adults survived after feeding on *Plasmodium* parasite positive blood until day 7 for *P. vivax* (**a**) and day 12 for *P. falciparum* (**b**) midgut dissection for oocyst detection (boxes show the 95% confedance interval).

The proportion of females surviving until the day of dissection for oocysts after feeding on *P. vivax* and *P. falciparum* positive blood was higher among adults emerged from larvae fed on *Z. mays* pollen diet than other diets (Fig. 6).

The proportion of *Plasmodium* oocysts found in *An. arabiensis* following an infected blood meal varied significantly depending on their larval diets (*P* < 0.05). *Anopheles arabiensis* adults emerged from larvae fed on *Z. mays* pollen showed the highest oocyst infection rate (45% for *P. vivax* and 27.5% for *P. falciparum*) followed by Tetramin fish food (30% for *P. vivax* and 20% for *P. falciparum*) (Table 2). There was no significant difference in *Z. mays* and Tetramin fish (*P* > 0.05). Those *An. arabiensis* fed on *T. latifolia* had infection rates of 22% for *P. vivax* and 8.3% for *P. falciparum*.

**Table 2:** The effect of larval diets on feeding efficacy, oocyst density and *P. vivax* sporozite infection rate of *An. arabiensis.*

Larval diet	No. of exposed	Feeding efficiency (%)	Adult survival (%)	No. dissected (+; %)	Oocyst/mid-gut ranges	ELISA tested (+)	CSP rate in % (95% CI)
*Z. mays*	320	87	238 (74.4)	120 (54; 45)	0–80	38 (5)	13.2 (4.4–28.1)
*T. latifolia*	320	83	148 (46.3)	120 (27; 22)	0–41	22 (1)	4.5 (0.5–22.8)
*P. juliflora*	320	81	150 (46.9)	120 (6; 5)	0–5	11 (0)	0
Tetramin fish	320	90	224 (70)	120 (36; 30)	0–77	33 (3)	9.1 (1.9–24.3)
Overall	1280		760	480 (123; 25.6)		104 (9)	8.6 (4.0–15.8)

**Figure 7:**
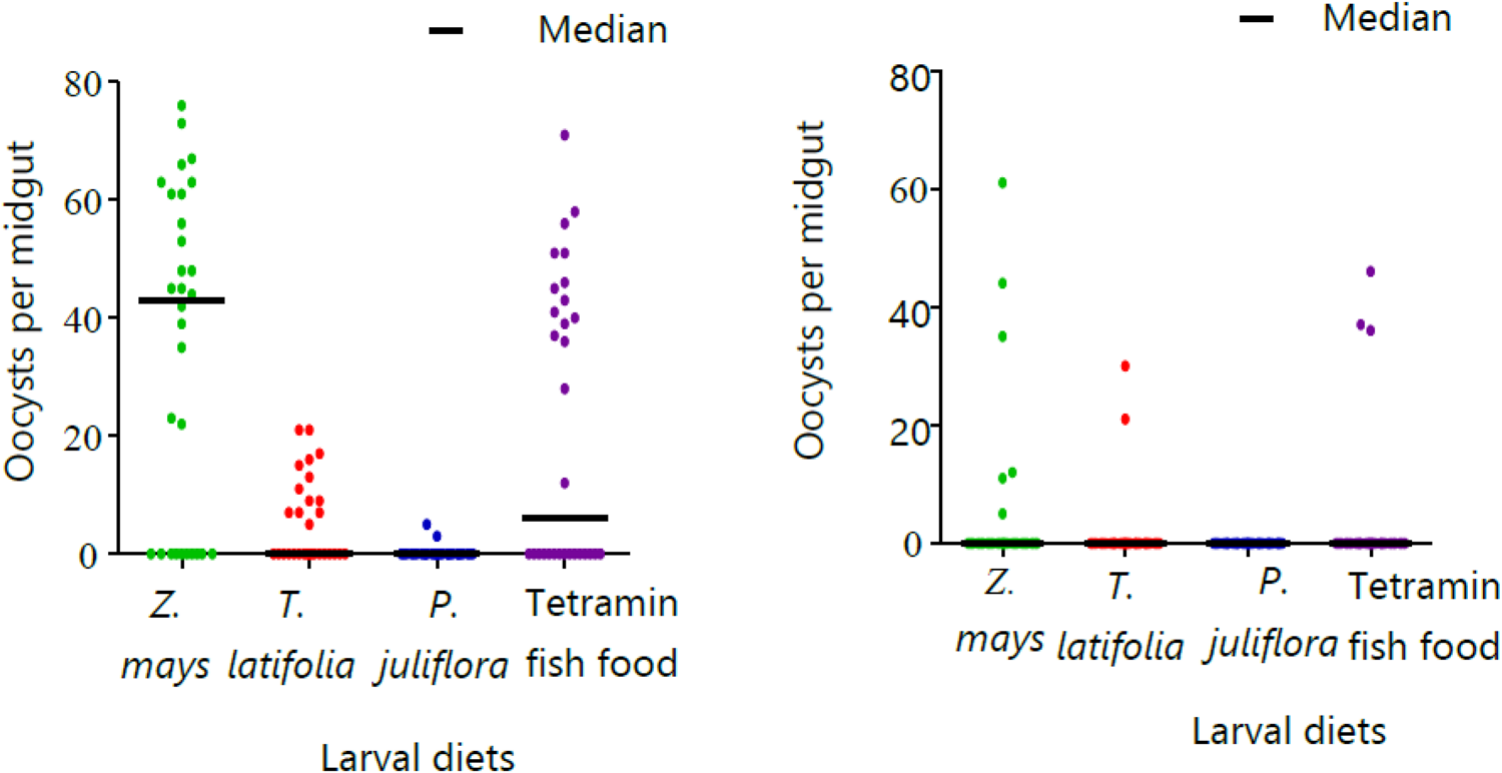
Median number of oocysts of *P. vivax* (left) and *P. falciparum* (right) in the mid-gut of female *Anopheles arabiensis* emerged from different larval diets.

The median number of *P. vivax* oocysts was higher in adult *An. arabiensis* emerged from larvae reared on *Z. mays* pollen diet (Fig. 7). The intensity of infection of *An. arabiensis* reared on *Z. mays* varied significantly from other pollen diets (*P* < 0.0001). The highest median number of oocysts was observed in *An. arabiensis* reared on *Z. mays* (median ꞊ 43) for *P. vivax* and (median = 0) for *P. falciparum* followed by Tetramin fish (median = 6) for *P. vivax* and (median = 0) for *P. falciparum.* The lowest mean number of oocysts was reported in those *An. arabiensis* reared on *P. juliflora* pollen larval diets (mean = 0.26, 1.04 ± SD, median = 0) for *P. vivax* and no infection for *P. falciparum*.

### Larval diet impacts on CSP infection rate

The overall CSP rate for *P. vivax* was 8.6% (95% CI 1.14–21.02; 9 of 104) (Table 2). *P. vivax* CSP rate of *An. arabiensis* reared on *Z. mays* pollen was 13.2% (95% CI 4.4–28.1; 5 of 38) followed by 9.1% (95% CI 1.9–24.3; 3 of 33) for Tetramin fish. No *An. arabiensis* from larvae fed on *P. juliflora* was positive for *P. vivax*. No *An. arabiensis* were found to be positive for *P. falciparum* CSPs (Table 3).

**Table 3:** The effect of larval diet on feeding efficacy, oocyst density and *P. falciparum* sporozoite infection rate of *A. arabiensis.*

Larval diet	No. of exposed	Feeding efficiency (%)	Adult survival (%)	No. dissected (+; %)	Oocyst/mid-gut range	ELlSA tested (+)	CSP rate in %
*Z. mays*	320	82	188 (58.7)	120 (33; 27.5)	0–6	14 (0)	0
*T. latifolia*	320	79	156 (48.7)	120 (10; 8.3)	0–3	7 (0)	0
*P. juliflora*	320	77	84 (26.2)	120 (0; 0)	0	3 (0)	0
Tetramin fish	320	89	166 (51.9)	120 (24; 20)	0–5	11(0)	0
Overall	1280					35 (0)	0

## Discussion

The effects of plant pollen larval diets on adult emergence, female size, pupation length, larval and adult survival, and parasite infection with *P. falciparum* and *P. vivax* were examined in this study. Pollen from *Z. mays* enhances larval survival, adult emergence, and *Plasmodium* parasite infectivity. Adult *An. arabiensis* emerged from larvae fed on *Z. mays* pollen were more susceptible *to P. vivax* and *P. falciparum* parasites *and* had the highest mean number of *P. vivax* oocysts and the longest survival times. Adults that emerged from larvae fed *P. juliflora* pollen had the lowest infection rate.

The development, survival, and adult emergence rates of *Anopheles* larvae are all influenced by the quantity and quality of their diet^7^. In farmland-associated habitats, maize pollen grains had a positive correlation with larvae abundance^20^. The grasses including *T. latifolia* also determine the abundance of larval stages of *Anopheles* mosquitoes and adult productivity^21^. *Prosopis juliflora,* an invasive plant, has been found to increase the number of *Anopheles* mosquitos^8^. There is evidence that maize pollen has beneficial effects on mosquito longevity, vector density, and larval development^5^. Additionally, the odour of maize pollen is utilised to attract gravid female malaria mosquitoes to lay their eggs^6^. This study investigated the effects of larval diets on survival of adults until oocyst development, the density of oocyst infections, and Plasmodium parasite susceptibility using the artificial membrane feeding experiment.

In this study, a substantial difference in larval survival rate was observed between larvae fed on different pollen diets. The pollen of *Z. mays* increased the survival of malaria mosquito larvae and adults. It has been demonstrated that larvae fed *Z. mays* pollen survived better^21^. In contrast, larvae fed *T. latifolia* pollen took less time to pupate than *Z. mays*, but those fed *P. juliflora* pollen diet took longer to mature into pupae. The ratio of carbon to nitrogen in the diet mat influence the growth and survival of the *An. arabiensis*^22^. The protein content of *Z. mays* pollen may be the most important element impacting An. arabiensis larval development and survival^5^. The larvae need enough food for ecdysis in order to avoid having a high mortality rate due to a prolonged larval period caused by a poor diet^23^.

When compared to the other pollen diets, larvae fed Z. mays pollen produced the largest female adults, which is proportional to the length of its wing. Numerous studies have demonstrated that one could produce small, intermediate, and large-sized mosquitoes by varying the amount and type of food^24^. This could be due to the nutritional values. An earlier study has shown that adult mosquitoes may grow to reasonably large sizes when their dietary needs are met^25^. Also, larger *An. arabiensis* live longer and lay more eggs than smaller *An. arabiensis*^26^.

Adult *An. arabiensis* emerged from larvae fed *Z. mays* pollen were more permissive to infection with *P. vivax* and *P. falciparum* parasites, and had the highest number of *P. vivax* oocysts. Adult body size, as well as the larval quality diets, may explain this^7,25^. The nutrient content of *Z. mays* may be superior to other diets and result in large-size female adults. Larger *Anopheles* mosquitoes ingest more blood meals, which correlate positively with the number of oocyst development^7,27^. Larger infected mosquitos have a better chance of surviving and may harbour more oocysts, resulting in the largest incidence of infective mosquitoes. In Ethiopia, communities that grow maize have been linked to higher rates of malaria infection^3^. Therefore, additional malaria vector control measures like larval source management could be strengthen in malaria endemic areas, particularly during the maize flowering seasons.

Additionally, it is possible that increasing the cultivation of crops like *Z. mays* could enhance the larval diet and perhaps generate favourable conditions for increased vector populations and transmission of malaria. This is because of the influence of larval nutrition on larval survival, adult fitness, oocyst density, and infection. Adult mosquitoes are the target for contemporary vector control. However, during a favourable transmission season, such as the growing season to maize, larval and pupal control efforts may be combined with adult control methods. Based on this study, some plants located close to breeding habitats can contribute to an increase in the number of vectors and the transmission of parasites since the food from these plants impacts the vector's capacity. In regions where maize is grown, intensified larval source management may be encouraged to reduce the number of mosquito larvae.

Although *An. arabiensis* from the wild were employed in the study, it was conducted in a laboratory setting; hence, field trials would be the next step. The failure to link the mechanisms of the various food source components to the vectorial components of malaria vectors was another limitation of the study. More study on this area is encouraged for the formulation of a tailored intervention.

## Conclusions

In conclusion, our results provide new insights into the effect of larval diet on mosquito larval survival, pupation, adult size and infectivity to *Plasmodium* parasites in Ethiopia. Of the diets, *Z. mays* pollen diet was superior to the other diets including the standard Tetramin fish food. Larvae fed on this diet produced adults with larger wing size and longer longevity, and greatly increasing the infectivity to malaria parasites. In contrast, the larval development time was significantly longer and mortality rate was higher when the larvae fed on *P. juliflora* pollen diet. These conditions also resulted in a reduction in adult longevity and adult fitness. Therefore, further investigations on the nutritional components of plant pollen as a source of mosquito larva diet, as well as the link between larval nutrition and parasite growth in vectors, should be conducted.

## Data Availability

The datasets used and/or analysed during the current study available from the corresponding author on reasonable request.
